# Impaired Chloroplast Biogenesis in *Immutans*, an Arabidopsis Variegation Mutant, Modifies Developmental Programming, Cell Wall Composition and Resistance to *Pseudomonas syringae*

**DOI:** 10.1371/journal.pone.0150983

**Published:** 2016-04-06

**Authors:** Gennady V. Pogorelko, Sekhar Kambakam, Trevor Nolan, Andrew Foudree, Olga A. Zabotina, Steven R. Rodermel

**Affiliations:** 1 Department of Genetics, Development, and Cell Biology, Iowa State University, Ames, IA, 50011, United States of America; 2 Laboratory of Functional Genomics, N.I. Vavilov Institute of General Genetics, Russian Academy of Sciences, Gubkina Street 3, 119991 Moscow, Russia; 3 Roy J. Carver Department of Biochemistry, Biophysics & Molecular Biology, Iowa State University, Ames, IA, 50011, United States of America; Hainan University, CHINA

## Abstract

The *immutans* (*im*) variegation mutation of Arabidopsis has green- and white- sectored leaves due to action of a nuclear recessive gene. *IM* codes for PTOX, a plastoquinol oxidase in plastid membranes. Previous studies have revealed that the green and white sectors develop into sources (green tissues) and sinks (white tissues) early in leaf development. In this report we focus on white sectors, and show that their transformation into effective sinks involves a sharp reduction in plastid number and size. Despite these reductions, cells in the white sectors have near-normal amounts of plastid RNA and protein, and surprisingly, a marked amplification of chloroplast DNA. The maintenance of protein synthesis capacity in the white sectors might poise plastids for their development into other plastid types. The green and white *im* sectors have different cell wall compositions: whereas cell walls in the green sectors resemble those in wild type, cell walls in the white sectors have reduced lignin and cellulose microfibrils, as well as alterations in galactomannans and the decoration of xyloglucan. These changes promote susceptibility to the pathogen *Pseudomonas syringae*. Enhanced susceptibility can also be explained by repressed expression of some, but not all, defense genes. We suggest that differences in morphology, physiology and biochemistry between the green and white sectors is caused by a reprogramming of leaf development that is coordinated, in part, by mechanisms of retrograde (plastid-to-nucleus) signaling, perhaps mediated by ROS. We conclude that variegation mutants offer a novel system to study leaf developmental programming, cell wall metabolism and host-pathogen interactions.

## Introduction

Variegated plants provide an excellent system to gain insight into mechanisms of chloroplast biogenesis and nuclear-organelle communication [1, 2, 3]. These plants have green- and white- (or yellow-) sectored leaves due to mutations in nuclear, chloroplast or mitochondrial genes. Whereas cells in the green sectors resemble wild type with respect to morphology and pigment composition (carotenoids and chlorophylls), plastids in cells of the white sectors lack pigments and organized lamellae. Over the years our research has focused on nuclear gene-induced variegations in which the cells have a uniform (mutant) genotype. This is in contrast to variegations in which the green sectors have a wild type genotype and the white sectors a mutant genotype [2].

One of the best-studied nuclear gene-induced variegations is the Arabidopsis *immutans* (*im*) mutant [4–6]. This mutation defines the gene for PTOX (plastid terminal oxidase), a plastid membrane enzyme that transfers electrons from plastoquinol (PQH_2_) to molecular oxygen, generating water and plastoquinone (PQ) [7–8]. PTOX activity is important for proper thylakoid development (chloroplast biogenesis), as well as for optimal functioning of mature photosynthetic membranes [5]. During early chloroplast biogenesis, when the PETC is not yet fully-assembled and functional, PTOX activity is the primary (and perhaps only) way to oxidize the PQ pool, e.g., in response to overreduction that might occur as a byproduct of redox reactions that utilize PQ as a substrate [9]. A striking example of this is the requirement for PTOX as a cofactor of the two, successive desaturation reactions of carotenoid biosynthesis, mediated by phytoene desaturase (PDS) and zeta-carotene desaturase (ZDS). A hallmark of chloroplast biogenesis is the progressive accumulation of carotenoids, and in the absence of PTOX, phytoene (the colorless substrate of PDS) accumulates because electron transfer into the PQ pool is inhibited, presumably because transfer into an overreduced pool is energetically unfavorable and/or that PQ becomes a limiting substrate for PDS as the PQH_2_/PQ ratio of the membrane increases [10–11]. Regardless of the precise cause, one consequence of phytoene accumulation is that carotenogenesis is blocked at the PDS step, thus preventing the accumulation of downstream, colored (photoprotective) carotenoids. This results in photooxidation of the contents of the developing organelle—the principle cause of white sector formation in *im* [11].

Under conditions of steady-state photosynthesis, PTOX is not able to compete with P700+ as an electron acceptor when mature leaves of wild type Arabidopsis are compared to *im* or to transgenic plants that overexpress PTOX [12]. Other studies, however, have established that PTOX does indeed play a role in mature chloroplasts: a) it serves as the terminal oxidase of chlororespiration (oxidation of the PQ pool in the dark) [13–14]; it helps balance the redox state of the PQ pool during cyclic electron flow (CEF) around photosystem one (PSI) [15–16]; and it acts as a photosynthetic safety valve during conditions of stress. As a safety valve, PTOX dissipates excess electron flow and thereby prevents the formation of high excitation pressures (1-qP, a relative measure of the reduction state of Q_A_) that would otherwise promote photooxidative damage and photoinhibition [5, 17].

Whereas photooxidation is likely the primary cause of white sector formation in *im*, the question arises why the green sectors have normal-appearing chloroplasts, despite lacking PTOX; all *im* alleles isolated to date are null [5]. Our current working hypothesis is that *im* variegation is governed by membrane redox and excitation pressures (EPs) during the early stages of chloroplast biogenesis, when thylakoid membranes are elaborated from precursor plastids, i.e., from etioplasts in dark-grown seedlings or from proplastids in meristems [11]. According to the ***threshold hypothesis***, developing *im* plastids with above-threshold redox states (overreduced, high EPs) become photooxidized, while plastids with below-threshold redox states (low EPs) develop into normal chloroplasts [5, 10]. Support for this hypothesis comes the observation that plastids do not have intermediate phenotypes, and that cells in the white sectors are heteroplastidic, i.e., they contain rare, normal-appearing chloroplasts in addition to the white plastids, which have a uniform size (decreased) and morphology. This indicates that *im* plastids are capable of developing independently from one another and display *plastid autonomous* behavior [10]. We surmise that this independence is a reflection of unique plastid biochemistries (and redox states) caused by factors such as the non-uniform distribution of photosynthetic substrates (light and CO_2_) across the leaf [18].

One assumption of our model is that the chaotic pattern of *im* variegation in the mature leaf is largely a reflection of the pattern of dicot leaf development, during which nearly all chloroplast differentiation and division processes occur in the leaf primordium (conversion of proplastids to chloroplasts), with subsequent development involving primarily cell and leaf expansion [19–20]. If this is the case, we hypothesize that the phenotypes of *im* plastids (green or white) are generally determined early in the leaf differentiation process, and that plastids do not change from white to green (or vice versa) in the expanding leaf. One purpose of the present research was to test this hypothesis.

Another purpose of the current investigations arose from early observations that the green leaf sectors of *im* have higher than normal photosynthetic rates and anatomies reminiscent of leaves adapted to growth in high-light conditions [21–22]. The white leaf sectors, on the other hand, have a normal thickness, although their palisade cells fail to expand. Accompanying these changes, the white sectors have downregulated expression of nuclear genes for plastid proteins involved in photosynthesis, and both tissue types have upregulated expression of genes for oxidative stress [23]. There are also tissue-specific alterations in the expression of genes that mediate source-sink interactions. These changes are consistent with experiments showing that the green sectors feed the white ones via apoplastic movement of sucrose from the green cells to the white ones, where it is hydrolyzed by invertase to yield products that can be utilized for energy [22].

In addition to genes for photosynthesis, oxidative stress and sink-source interactions, the global transcriptomic analyses revealed that the green and white sectors have dramatic tissue-specific alterations in the expression of genes for cell wall biosynthesis [22–23]. This suggests that cell wall remodeling plays a central role in the biochemical, physiological and morphological adaptions of each tissue-type to an absence of PTOX. Interestingly, these changes were also accompanied by modified patterns of expression of genes for pathogen response. Therefore, the second aim of our research was to gain insight into the poorly-understood cell wall determinants that promote pathogen susceptibility and response by determining the composition of cell walls in *im* green and white sectors, and second, by examining the response of these tissue-types to infection by the pathogen *P*. *syringae*.

## Materials and Methods

### Plant materials and growth condition

Arabidopsis plants with the *spotty* allele of *im* [15] were germinated and grown at 22°C under continuous illumination, first three weeks at 15 *μ*mol m^−2^s^−1^ for 5 days, then at 50–60 *μ*mol m^−2^s^−1^ for the rest of their growth. *Col-0* served as wild type controls.

### Determination of protein and RNA

Leaves were harvested from 7-week-old Col-0 and *im* plants (green and white sectors) and weighed. These tissues were used for both protein and RNA determinations as described previously [22–23]. For the protein analyses, leaf samples were frozen in liquid nitrogen, homogenized and resuspended in extraction buffer [10 mM NaCl; 10 mM MgCl2; 5 mM EDTA; 10 mM β-mercaptoethanol; 25 mM Tris-HCl (pH 7.5); and 1 mM PMSF]. After centrifugation (5,000g for 15 minutes), equal fresh weights of the protein extracts were subjected to 10% SDS-PAGE analysis, followed by Coomassie Blue staining (Fisher Scientific).

Total RNA was extracted using the Trizol Isolation kit (Invitrogen). The RNA samples were then loaded onto denaturing gels on a fresh weight basis; the gels contained 1% agarose and 3% formaldehyde in 10 mM phosphate buffer (pH 7.0). Following electrophoresis, the gels were stained with ethidium bromide.

### cDNA preparation and quantitative real-time PCR

Total RNA was assessed on a 0.7% agarose gel, quantified and normalized by NanoDrop ND-1000 spectrophotometer. For qRT-PCR analysis, first-strand cDNAs were synthesized from 3 μg of total RNA using Superscript III reverse transcriptase (Invitrogen) according to the manufacturer's recommendations.

Real-time qPCR was performed on an iCycler Real-Time system (Bio-Rad). Each reaction was done in a final volume of 25 μl containing 12.5 μl of SYBR Green Master Mix reagent (Fermentas), 300 ng of cDNA (for gene expression analysis) or 200 ng of total DNA (for chloroplast DNA quantification) sample, and 200 nM gene-specific primers (S1 Table), designed to generate products of 150–200 bp using Vector NTI 9.0 software (Informax). The qPCR conditions were as follows: 50°C for 2 min, 95°C for 10 min, 45 cycles of 95°C for 15 s, 60°C for 30 sec, and 72°C for 45 sec. At the end of the 45 cycles, a melting curve was generated to analyze the specificity of the reactions. Each sample was tested in three technical replicates. Expression level of the Actin 2 gene was used as the endogenous control. The relative expression level or chloroplast DNA concentration was calculated as 2^–ΔΔCT^ [ΔC_T_ = C_T_, _gene of interest_ − C_T, Actin2_. ΔΔC_T_ = ΔC_T, treatment_ − ΔC_T, Col-0_]. Absolute numbers were calculated as described previously (Lu et al., 2012).

### Histochemical procedures

#### Cell viability

Cell viability was monitored using the Evans Blue [24]. For this assay, excised leaflets from *im* and *Col-0* (7-week-old) were vacuum infiltrated with 0.1% (w/v) Evans blue (Sigma-Aldrich) for 10 min, then destained by washing three times with distilled water. The leaves were observed with a Zeiss Axioskop 2 plus (Carl Zeiss) microscope. In this assay, necrotic cells are stained, but living cells exclude the dye.

#### Chloroplast DNA

Chloroplast DNA was visualized by confocal microscopy of DAPI-stained tissues [25]. DAPI (4',6-diamidino-2-phenylindole) is a fluorescent dye that has high affinity for AT-rich regions of DNA. For this procedure, green and white segments were dissected from 7-week-old *im* and *Col-0* leaves, fixed in 2.5% (v/v) glutaraldehyde, then stained for 30 min with 1 ug/mL DAPI. The destained samples were examined with a Carl Zeiss LSM 510 META confocal microscope, using 440 nm excitation and 685 nm emission wavelengths for chlorophyll and 410 nm excitation and 460 nm emission wavelengths for DAPI.

#### Determinations of plastid number

To measure plastid numbers, light microscopy was performed on toluidine blue or Schiff’s reagent stained sections from fixed 4 and 8 week-old *im* and *Col-0* Arabidopsis using previously described procedures [26]. Toluidine blue is a basic dye with high affinity for acidic components (DNA, polysaccharides) [27] and Schiff’s reagent preferentially binds to the starch [28]. In brief, leaf segments (3 to 5 mm^2^) were incubated for 3 h in 1.6% (w/v) paraformaldehyde and 0.2% (w/v) glutaraldehyde in 25 mM sodium phosphate buffer (pH 7.1), washed twice with water (15 min each), and dehydrated in a graded ethanol series (20 min at each step). The dehydrated samples were moved to 4°C and then gradually infiltrated with cold LR White embedding resin (Fisher Scientific), first in 33% (v/v) then 66% (v/v) resin in 100% ethanol (24 h for each infiltration), and finally in 100% resin (three times, 24 h each time). The infiltrated samples were transferred to gelatin capsules containing 100% resin for embedding, and the resin was polymerized by incubation at 65°C for 20 h. Sections of 300 nm were prepared using a Leica VT1000S vibratome, stained either with 0.05% toluidine blue or Schiff's reagent, and visualized with a Zeiss Axioskop 2 plus (Carl Zeiss) microscope. Images were captured with a Nikon DXM 1200 digital camera, and plastid numbers were counted using ImageJ software available from the NCBI website.

#### Callose deposition

To examine callose deposition, expanded leaves from six independent 7-week-old plants were cleared with 96% ethanol and fixed in an acetic acid:ethanol (1:3) solution for 2 hours. The samples were incubated sequentially for 15 min each in 75% ethanol, 50% ethanol, and 150 mM phosphate buffer (pH 8.0), then stained for 2 h at 25°C in 150 mM phosphate buffer (pH 8.0) containing 0.01% (w/v) aniline blue. After staining, the leaves were examined by UV fluorescence microscopy using a Leica DMIRE2 microscope.

#### Reactive oxygen species

For hydrogen peroxide visualization, leaves were cut from 7-week-old plants and dipped for 16 hours in a solution containing 1 mg/mL DAB (3,3’-diaminobenzidine), pH 5.0. Chlorophyll was extracted with hot 96% ethanol, and the leaves were photographed with a Canon EOS300D camera.

### Transmission electron microscopy (TEM) and chlorophyll fluorescence

Leaf tissues were fixed in a solution of 2% glutaraldehyde and 2% paraformaldehyde in a 0.1 mM cacodylate buffer (pH 7.2). Subsequent steps were performed as described previously [29]. Images were captured on a JEOL 2100 STEM. Chlorophyll fluorescence was observed with a Carl Zeiss LSM 510 META confocal microscope using 440 nm excitation and 685 nm emission wavelength for chlorophyll.

### Cell wall composition analyses

Cell walls were isolated from plant tissues as described by Zabotina et al. [30]. Cell wall extracts were prepared separately from white *im*, green *im*, and *Col-0* wild type leaves. Monosaccharide compositions were determined as described by Pogorelko et al. [31].

To assess lignin contents, cell wall extracts (3 mg) were treated with 800 uL of fresh acetyl bromide in acetic acid (1:16) for 2 hours at 50°C. An aliquot (15 uL) of each sample was then mixed with 85 uL of acetic acid containing 0.05M sodium hydroxide and 0.03M hydroxylamine hydrochloride. UV absorbance was measured at 280 nm to estimate lignin contents.

To determine cellulose contents, cell wall extracts (10 mg) were suspended in 3 mL of a nitric-acetic acid mixture (545 uL water, 2.2 mL glacial acetic acid, 255 uL of 16 M nitric acid) and heated at 100°C for 30 minutes. After incubation, the solid phase was sedimented by centrifugation and washed 3 times with 3 mL of deionizer water, once with 3 mL of 100% acetone, and then dried at 50°C for 48 hours. Dried residues were weighed to measure cellulose content.

### Infection assay, *in planta* growth and disease development

Cultures of *Pseudomonas syringae* DC3000 were grown overnight in LB medium at 25°C up to OD_600_ = 1. Cells were collected by centrifugation at 4000g for 10 minutes and re-suspended in infiltration buffer (10 mM MES, 10 mM MgCl_2_, pH = 5.8) at a concentration of 10^4^ cfu/mL; the resuspended cells were pressure-infiltrated into the abaxial side of leaves. To assay bacterial growth, a circle of 7 mm in diameter was cut from the leaf, ground in 500 uL of a 10 mM MgCl_2_ solution, and a series of dilutions (from 10^−1^ up to 10^−8^) were plated on LB media containing 50 mg/L of rifampicin. Calculations were performed using the dilution that gave approximately 50–70 colonies.

## Results

### The threshold hypothesis: plastid composition of sectors is fixed early in leaf development

**Fig 1:**
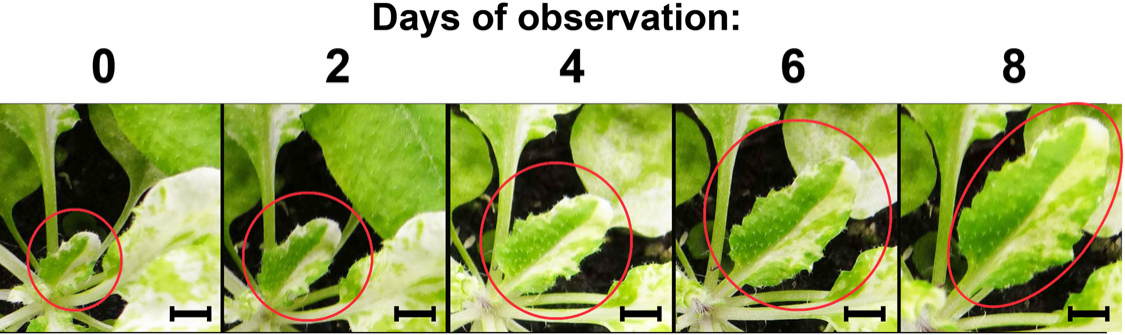
Sector identity is maintained during im leaf expansion. Images of the same *im* leaf were captured by light microscopy every two days from early expansion (day 0) to the attainment of full expansion (day 8). Bar = 5 mm.

One element of the threshold hypothesis that warrants examination is the assumption that the pattern of variegation in mature *im* leaves is primarily a reflection of dicot leaf development. According to this scenario, each proplastid in an *im* meristem cell differentiates into either a chloroplast or a white plastid depending on its redox state, then divides to generate a clone of plastids and cells (representing a sector) in the leaf primordium. Consistent with this model, green and white sectors have been observed in *im* leaf primordia by confocal microscopy [6]. Fig 1A further reveals that sector identity does not change during leaf expansion phase; sectors merely increase in size.

This suggests that plastid identity and sector boundaries become fixed during the primordium stage, and that white plastids do not differentiate into chloroplasts (or vice versa) during or after the expansion phase. One reason for the relatively limited number of sectors in the mature leaf is that leaf development begins with a small number of proplastids and meristem cells (fewer than 20 proplastids per meristem cell,) and gives rise to a large number of cells and chloroplasts (100 or more chloroplasts in a typical mesophyll cell) [19–20]. Thus, early differentiation events that flip a plastid into one state or another can be magnified by division and expansion into a large sector in the mature leaf.

In contrast to the above generalization, we note that the white sectors of mature *im* leaves occasionally have very small green spots that correspond (via UV and light spectroscopy) to heteroplastidic cells or to a small clone of chloroplast-containing cells. We propose that these arise from the limited number of plastid and cell divisions that occur in expanding and mature leaves [19], perhaps as a consequence of the ability of dividing plastids to alter their developmental fate according ambient EP thresholds, as early suggested by Wetzel et al. [10] in the ***developmental race model of plastid differentiation***.

### The threshold hypothesis: im white sectors have sharply decreased plastid numbers

As described in the ***Introduction***, the green and white leaf sectors of *im* display alterations in biochemistry, physiology and morphogenesis caused, in part, by differential reprogramming of gene expression in the two tissue types [21–23, 32]. This reprogramming results in the acquisition of tissue-specific strategies to cope with oxidative stress, as well as alterations that promote sink-source interactions between the green (source) and white cells (sinks). As part of an ongoing effort to gain insight into these processes, the present studies sought to examine plastid numbers in the white sectors. These experiments were prompted by the chance observation of relatively few plastids in some cells of these sectors.

**Fig 2:**
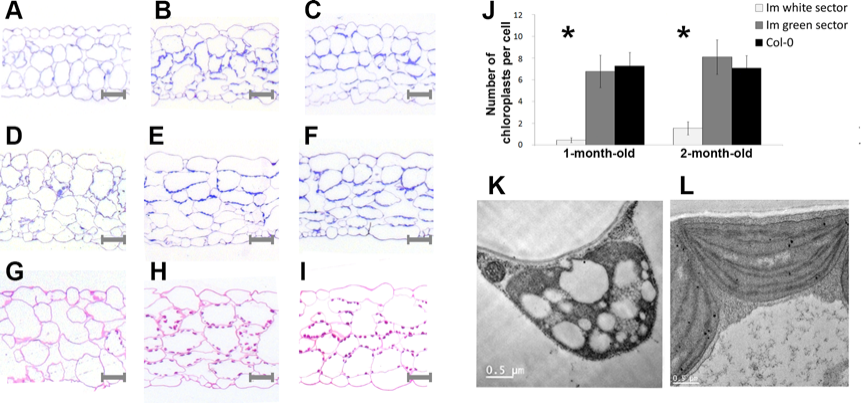
Tissue anatomy and plastid numbers. Leaves from 4- and 8-week-old Col-0 and *im* (white and green sectors) were fixed, stained and examined by light microscopy. (A, D, G) *im* white; (B, E, H) *im* green; (C, F, I) Col-0; (A, B, C) 1 month-old; (D–I) 2 month-old. (A-F) Sections were stained with toluidine blue and plastids were counted using Image J software (NCBI website); (J) the sections were 500 nm thick and approximately 150 cells were analyzed for each tissue-type. Asterisks indicate significant difference (t-test, p < 0.01). (G-I) Sections from 2-month-old leaf tissues were stained with Schiff’s reagent for starch. TEM images of representative plastids from fully-expanded *im* white (K) and wild type leaves (L).

For these analyses, we counted plastid numbers in toluidine blue-stained sections of fully-expanded leaves from 4- and 8-week-old plants growing under continuous light; the samples included wild type leaves, and tissues dissected from *im* green and white sectors (Fig 2).

In terms of gross anatomy, the data are consistent with earlier observations [21] showing that the green sectors (Fig 2B, 2E and 2H) have enlarged air spaces and larger than normal mesophyll and epidermal cells, whereas the white sectors (Fig 2A, 2D and 2G) have smaller than normal cells and a lack of palisade cell expansion. Ultrastructural analyses reveal that chloroplasts in the *im* green and wild type tissues have normal morphologies (Fig 2L), whereas plastids in the white sectors are highly vacuolated, lack lamellae and are much smaller than normal (Fig 2K); the TEM data confirm previous findings [10].

Fig 2J reveals that the toluidine blue-stained *im* green sectors have wild type chloroplast numbers per cell at both developmental time points, while plastid numbers in the white sectors are dramatically reduced 5- to 10-fold, with many sections lacking a plastid. The three tissue-types were also stained with Schiff’s reagent (specific for starch) as an alternate way to estimate plastid numbers (Fig 2G, 2H and 2I). The light micrographs show that cells in both green tissues have an abundance of starch-containing bodies (i.e., chloroplasts) in their peripheries, consistent with the quantitative analyses (Fig 2J). As expected, the white tissues are not stained because they are not photosynthetic and do not accumulate starch (Fig 2G). Considered together, the data in Fig 2 indicate that *im* white sectors have drastic reductions in plastid number and size, compared to wild type or *im* green sectors. These reductions could be caused by decreased rates of plastid division and/or by decreased plastid viability.

### The threshold hypothesis: chloroplast DNAs are amplified in *im* white sectors

**Fig 3:**
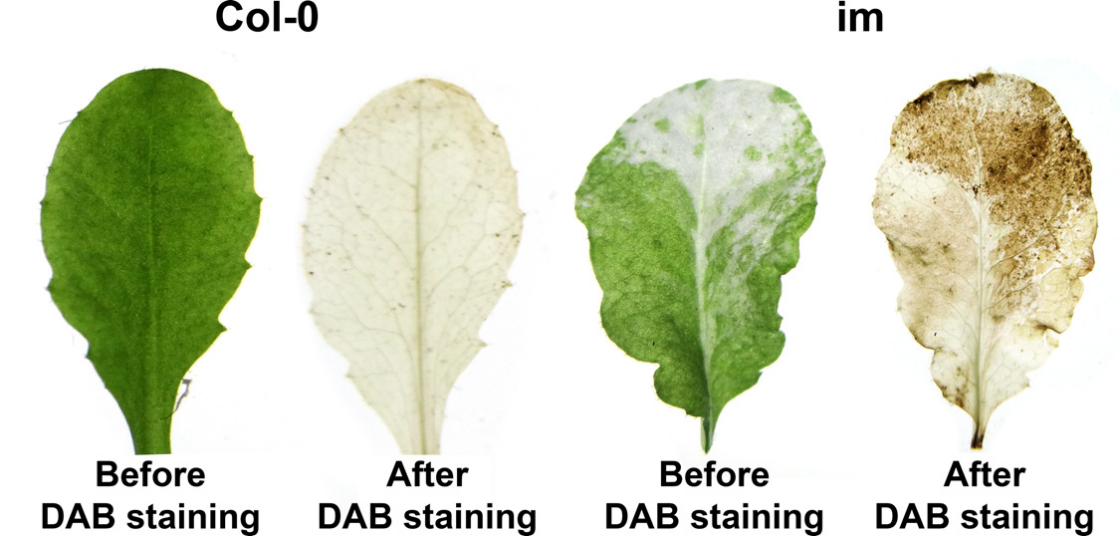
ROS accumulation. Leaves were detached from 7 week-old Col-0 and *im* and stained with DAB, specific for H_2_O_2_, by methods described in the Materials and Methods.

To address this question, we first monitored ROS accumulation in *im* and wild type leaves by staining the tissues with DAB (3′, 3′- diaminobenzidine), a reagent that detects H_2_O_2_. Fig 3 shows that *im* white sectors stain strongly with DAB, compared to wild type leaves, which are not stained.

This observation confirms previous findings that white, but not green, *im* sectors generate ROS [33]. This raises the possibility that ROS accumulation, either directly or indirectly, affects plastid viability, division and/or size.

**Fig 4:**
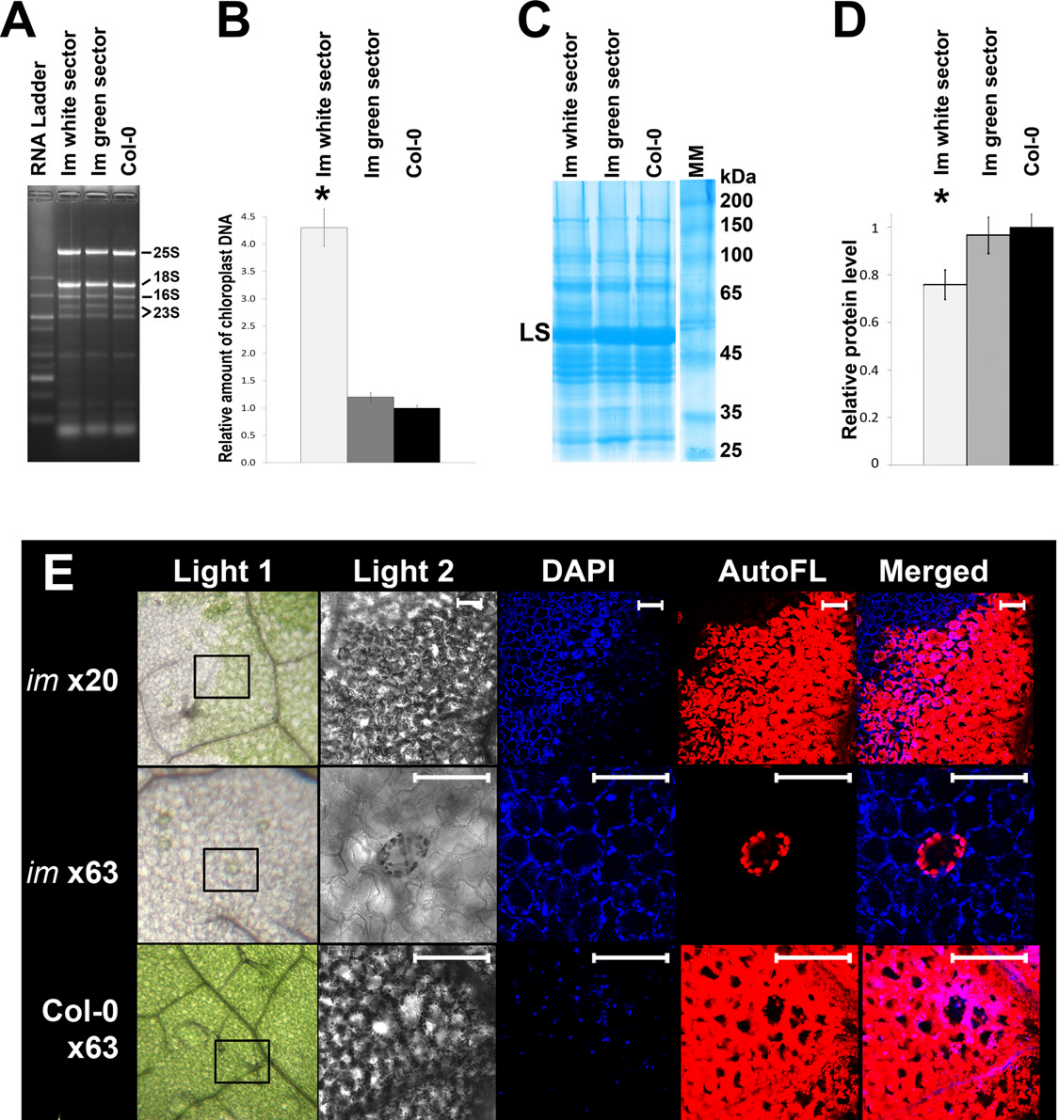
RNA, protein and chloroplast DNA. (A) Ethidium bromide-stained denaturing gel of total cell RNAs extracted from 7 week-old wild type Col-0 and im leaves (white and green sectors). The RNAs were loaded on the gel on a fresh weight basis. The rRNA bands are labeled. (B) Chloroplast DNA-specific primers. Real-time qPCR revealed 4 times the amount of chloroplast DNA in white tissues compared to green and wild-type tissues. Same samples as in (A). (C) Coomassie blue-stained 12.5% SDS-polyacrylamide gel of total cell proteins using the same samples as in (A). The proteins were loaded on a fresh weight basis. The large subunit (LS) of Rubisco is indicated; it comprises nearly all of the band migrating at 55 kDa. (D) Protein amounts in the gel lanes were quantified using ImageJ software. Asterisks indicate a significant difference (t-test, p < 0.05). (E) Confocal fluorescence microscopy. Leaves from 2 month-old *im* and Col-O were fixed and stained with DAPI (1 ug/ml), then certain leaf area (indicated on Light 1) examined by confocal microscopy (Light 2, DAPI, AutoFL, and Merged, as described in the Materials and Methods. Auto FL is chlorophyll. Bar = 20 μm.

We next examined profiles of total cell RNA and total cell protein accumulation in expanded leaves from *im* (dissected green and white sectors) and wild type. Fig 4A shows that all three tissue-types have similar RNA profiles with respect to their rRNA binding patterns.

These include the cytosolic 80S rRNAs (25S and 18S) and the plastid 70S rRNA species (16S rRNA and the two 23S rRNA breakdown products). All of these rRNAs are present in roughly equal amounts in the three samples. The three samples also have similar total cell protein profiles, as monitored by SDS PAGE analysis (Fig 4C), with the exception that protein abundance is ~20% reduced in the white sectors (Fig 4D). This reduction was evident when the gels were loaded on an equal fresh weight basis (as in Fig 4C and 4D) or on an equal protein basis. At least part of this decrease can be attributed to Rubisco, as indicated by the decreased intensity of the LS band; Rubisco can constitute up to ~50% of the total cell protein in Arabidopsis leaves [34].

Taken together, the data in Figs 2 and 4 indicate that sharp reductions in plastid number and size in the white sectors are not matched by similar reductions in plastid ribosome concentrations and protein biosynthetic capacities. To examine why this might be the case, we considered the possibility that chloroplast DNA is amplified in the white sectors. To test this hypothesis, we first performed confocal fluorescence microscopy on *im* and wild type leaves stained with DAPI (4',6-diamidino-2-phenylindole), a DNA-specific fluorescent dye (Fig 4E). Fig 4E reveals that DAPI-stained white sectors fluoresce more intensely than *im* green or wild type tissues, suggesting that they have more chloroplast DNA. To confirm this, we examined the three tissue types by Real-Time qPCR, using primers specific for chloroplast DNA. Fig 4B shows that the amount of chloroplast DNA relative to the amount of genomic DNA is similar in the wild type and *im* green samples, but amplified about 5-fold in the white sectors.

In summary, the data in Figs 2 and 4 are consistent with the idea that chloroplast RNA and protein accumulation are significantly different from normal on a per plastid basis, perhaps because mechanisms of plastid DNA amplification are deployed in cells of white sectors.

### Cell wall remodeling in *im*

**Fig 5:**
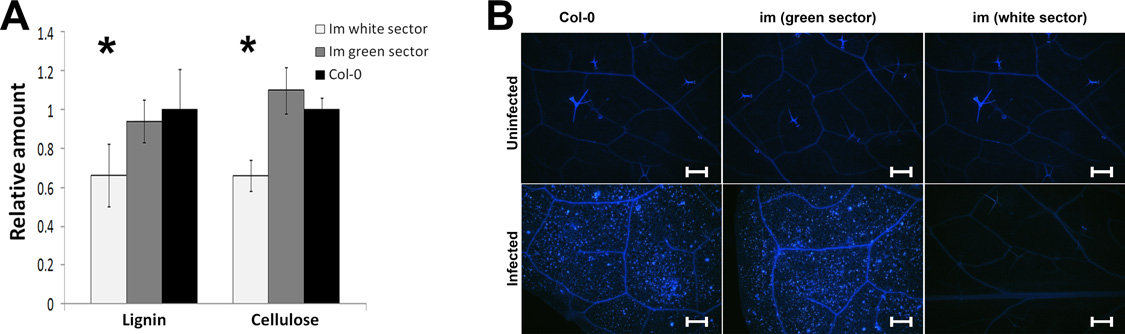
Composition of cell wall polymers. (A) Lignin and cellulose contents in the cell walls of Col-0 and *im* (white and green sectors). The contents were determined on a per mg basis of cell wall extracts. (B) Callose accumulation in Col-0 and *im* (white and green sectors) before and after infection with *P*. *syringae*. Expanded leaves from 7 week-old plants were cleared with ethanol and stained with aniline blue. The images were captured via UV fluoresecence microscopy. Bars = 200 μm.

As described in the **Introduction**, the green and white *im* sectors display striking, tissue-specific alterations in the expression of genes whose products play a role in cell wall metabolism. This reprogramming likely reflects a whole plant growth strategy that involves, primarily, the optimization of tissue-specific responses to oxidative stress, as well as interactions between the green and white cells that promote their ability to serve as sinks (white cells) or sources (green cells) [22, 23]. One of the purposes of the present research was to test this hypothesis and investigate cell wall compositions of the *im* green and white sectors. In Fig 5A we assayed cell wall extracts from *Col-0* and *im* (white and green sectors) for their content of lignin and cellulose -the two predominant polymers in cell walls.

The data show that the relative amount of each polymer is similar in both green tissues, but that each is much reduced in the *im* white sectors. The monosaccharide composition of other cell wall polysaccharides is also similar in each of the two green tissues, but altered in cell walls from the white sectors (Table 1).

**Table 1:** Monosaccharide composition (mol%) of cell wall from immutans and *Col-0* rosette leaves.

	Fuc	Rha	Ara	Gal	Glc	Xyl	Man	GalA	GlcA
*Col-0*	2.37±0.02	6.44±0.37	9.61±0.97	23.08±0.26	33.10±1.71	18.12±0.51	5.93±0.03	0.40±0.05	0.81±0.07
*im* (white sector)	2.10±0.04*	5.69±0.19	7.08±0.48	15.01±1.59*	50.61±4.50*	15.24±2.04	3.13±0.41*	0.39±0.03	0.74±0.05
*im* (green sector)	2.36±0.07	6.74±0.15	9.59±0.46	21.02±0.20	34.05±1.99	19.39±0.75	5.49±0.60	0.56±0.03	0.80±0.05

*Significantly different from wild type (t-test, p<0.01, n = 3)

Thus, while non-cellulosic glucose content is increased by 50%, the amounts of fucose (12%), galactose (35%), and mannose (47%) are reduced in the white sectors compared to the green sectors and *Col-O*. Taken together, the data in Fig 5A and Table 1 indicate that the cell walls of *im* white differ significantly from wild type, and that while cell walls of *im* green resemble those of wild type more closely, they are not the same.

### Cell wall remodeling in im: responses to Pseudomonas syringae infection

**Fig 6:**
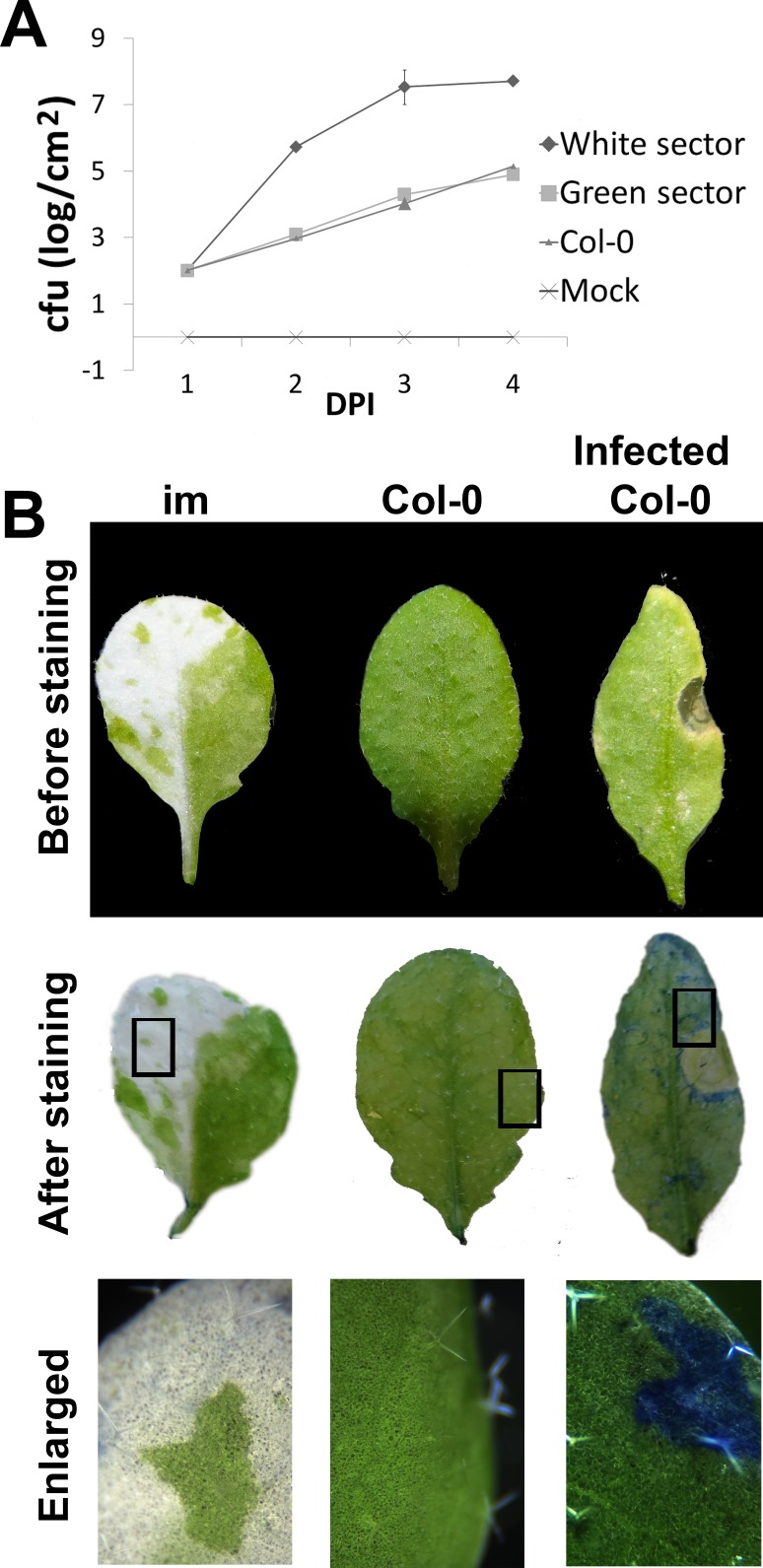
Evans blue exclusion test. (A) Leaves of *im* and Col-0 were infiltrated with a *P*. *syringae* cell culture at a density of 10^4^ colony forming units (cfu), and bacterial growth was monitored daily for 4 days after infection (DPI). (B) Leaves were detached from 7 week-old Col-0 and *im* and stained with Evans blue. In some experiments, detached wild type leaves were infiltrated with a *P*. *syringae* cell culture (density of 10^4^ cfu) prior to staining. In the Evans blue exclusion test, living cells exclude the dye, while dead cells take it up.

The next question we asked was whether the cell wall modifications in *im* affect pathogen response. To address this question, we analyzed susceptibility of *im* to *P*. *syringae* DC3000. This pathogen causes necrosis and cell death, as illustrated by Evans blue staining of infected tissues (Fig 6).

This dye is taken up by dead cells, but not living ones, as shown by control non-infected wild type leaves, which do not retain the stain. Non-infected *im* do not retain the stain either. This indiactes that the white *im* sectors are composed of living rather than dead cells, in contrast to necrotic cells and tissues that are found in the white sectors of some variegations [35].

To obtain a quantitative estimate of the susceptibility of *im* to *P*. *syringae*, bacterial cultures were infiltrated into the leaves of *im* and *Col-0* plants, and bacterial cell growth was monitored over the course of four days post-infection. Fig 6A shows there is a marked increase in susceptibility of the white tissues to the pathogen, while susceptibility of the green sectors resembles that of wild type. To investigate the source of these differences in susceptibility, we analyzed the expression of several diagnostic defense-related genes by Real-Time qPCR of RNAs from non-infected and infected leaves of *im* (green and white sectors) and wild type. The genes included MAPK*/ERK kinase kinase member A1* (*MEKK1*), *PHYTOALEXIN DEFICIENT 4* (*PAD4*), *ENHANCED DISEASE SUSCEPTIBILITY 1* (*EDS1*), *PATHOGEN RELATED 1* and *5* (*PR1*, *PR5*), and *β-GLUCAN SYNTHASE 2* (*bGS2*): *MEKK1* is involved in responses to ROS; *PAD4* and *EDS1* mediate responses to multiple stresses; *PR1* and *PR5* are well-known pathogenesis-related genes; and *bGS2* is involved in callose deposition.

**Fig 7:**
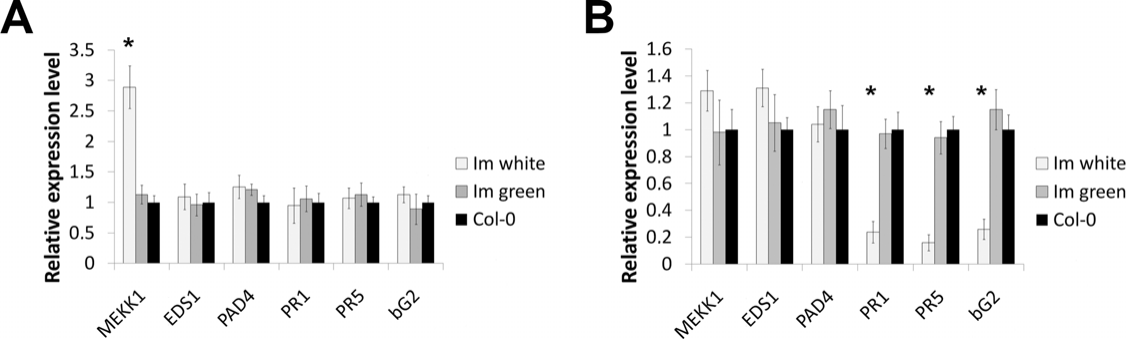
Expression of pathogen response genes. Real-time qPCR evaluation of pathogen-related genes involved in immune response in (A) non-infected and (B) infected plants at 4 DPI. The samples were as in Fig 6B, and the genes are described in the text.

Fig 7A and 7B show the results of the Real-Time qPCR experiments.

In these experiments we calculated the relative RNA amounts for each treatment (infected versus non-infected) across the six genes. Although these analyses preclude comparisons between treatments, it has been established that all six genes are sharply upregulated in infected versus non-infected wild type Arabidopsis leaves (see references above). Given these considerations, our experiments revealed that the six genes have similar levels of expression in non-infected wild type and *im* (white and green sectors), with the exception of a striking upregulation of *MEKK1* in *im* white sectors (Fig 7A). In the infected samples, it can be inferred that all six genes are significantly upregulated to a similar extent in both green tissues. *PR1*, *PR5* and *bG2* expression, on the other hand, are less responsive to the pathogen in the *im* white sectors. This correlates with the enhanced susceptibility of these sectors to *P*. *syringae* infection.

To examine this correlation, we monitored callose deposition in the three tissue types of infected and non-infected leaf tissues by aniline blue staining. Callose is a (1,3)-β-glucan polymer, and its induction in response to pathogen infection causes cell wall thickening and (perhaps) resistance to further infection [36]. Fig 5B shows that callose is not induced in any of the non-infected samples, but that upon infection, high levels of callose are induced in both *im* green and wild type. By contrast, there is no callose deposition in the white sectors. These observations are consistent with the idea that *im* white tissues are more susceptible to *P*. *syringae* than the green tissues because of defects in defense gene expression.

## Discussion

### White sectors and sink demand

One purpose of the present studies was to gain insight into the threshold mechanism of *im* variegation. Previous studies have demonstrated that the green and white *im* sectors differ significantly with respect to their morphology, biochemistry and physiology. Transcript profiling has indicated these differences are due, in part, to a reprogramming of development in the green and white sectors that optimizes whole plant growth [4, 21, 22, 23]. This reprogramming is strongly influenced by at least two factors, both of which occur very early in leaf development. The first involves tissue-specific adaptations to oxidative stress that are likely elicited in response to overreduced plastid membranes (the primary lesion in *im*). The second factor occurs in response to sector formation (in the leaf primordium) and involves the fashioning of the sectors as effective sources (green sectors) or sinks (white sectors).

Previous studies have revealed that the green sectors of *im* have a gross anatomy reminiscent of wild type leaves that have been adapted to growth in high light, despite growth of the mutants under low light (permissive) conditions [21]. We presume this morphology is a response to oxidative stress in the developing leaf. Cells in the green sectors also have elevated photosynthetic rates, perhaps as a consequence of sink demand; photosynthetic rates in source tissues are often feedback controlled by sink strength [22]. Although the reason for the increased rates is not understood, the present data show they are not associated with an increase in chloroplast number or size, both of which are similar to those in wild type leaf cells.

In contrast to the green sectors, plastid numbers (and sizes) are drastically reduced in cells of the white sectors. These reduction are not accompanied by significant cell and/or tissue necrosis, as revealed by Evans blue staining. Surprisingly, RNA amounts and protein synthetic capacities are only marginally affected in the white sectors, but chloroplast DNAs are dramatically amplified. These alterations are accompanied by reductions in cell size, confirming previous FACS analyses of protoplasts from *im* (white and green sectors) and wild type leaves [32]. Taken together, these observations suggest that white *im* tissues are fashioned into effective sinks during leaf development, and that this process involves 1) a decrease in plastid division and/or viability; 2) the maintenance of near-normal levels of protein synthetic capacity; and 3) an elevated potential for chloroplast gene expression (enhanced chloroplast DNA amounts).

Although mechanisms underlying sink demand are poorly understood, we suggest that the characteristics we have discovered play an important role in the growth strategy of the plant. We assume that this strategy attempts to maximize the potential of the green and white sectors to serve as sources (manipulation of source strength, e.g. via regulating photosynthetic capacities) or sinks (manipulation of sink demand, e.g., via regulating rates of plastid division) by calibrating their relative proportion throughout leaf development. It might be noted that the characteristics of *im* white sectors we have described are not universally applicable to white leaf tissues; e.g., the white sectors of some variegations have significant cell and tissue necrosis [35]. This indicates there are probably many different strategies to optimize growth and that these depend on responses to the primary lesion in the mutant, directly and indirectly.

In addition to serving as determinants of sink demand, the unique characteristics of *im* white sectors might be advantageous for another reason. It is interesting that plastids in the white sectors resemble etioplasts in dark grown seedling inasmuch as they contain nearly the full complement of proteins required for photosynthesis [37]. One reason for this is to prime the organelle for subsequent light development, thus avoiding a developmental delay that might be toxic for the developing chloroplast when mechanisms of light capture and use are still not fully operational and integrated. In an analogous manner, we propose that elevated chloroplast DNA levels and near-normal protein synthetic capacities are a strategy to poise the white plastid for further development. This could occur in cases where a white plastid divides and the progeny find themselves with redox thresholds compatible with normal chloroplast biogenesis; differentiation into chloroplasts could commence immediately. Alternatively, white and green *im* plastids are precursors of plastids that are transmitted to progeny via selfing or outcrossing, and during these developmental transformations (e.g., gametogenesis, seed and embryo development), an individual plastid undergoes a series of dedifferentiation and redifferentiation events. In fact, branches with nearly all white plastids can give rise to all-green plants in the next generation under permissive (low light) conditions [10, 38]. This suggests that maintenance of high protein synthetic capacities might help poise a white plastid for its conversion into another plastid type.

### Retrograde signaling and ROS

It is well-established that retrograde (plastid-to-nucleus) signaling pathways play an important role in coordinating the early events of chloroplast biogenesis [39–42]. We have also proposed that plastid signals relay the metabolic and developmental state of developing *im* plastids to the nucleus, where they elicit changes in gene expression that, in turn, modify plastid and cell phenotypes, as well as patterns of leaf development (termed **plastid developmental signals**) [3, 43]. While a number of retrograde pathways have been identified in chloroplasts, nearly all poorly-defined, one of the best-studied is elicited in response to alterations in chloroplast redox [44–48]. Our finding that developing *im* membranes have overreduced PQ pools suggests that one or more pathways responsive to redox are operational in developing *im* leaves [6, 11].

In addition to signals emanating from the redox state of the PQ pool, it might be noted that PTOX itself might be a generator of plastid signals. This possibility comes from studies showing that ROS are produced as a side product of the PTOX reaction in liposomes containing purified PTOX; in *E*. *coli* membranes that contain PTOX; in tobacco PTOX overexpression plants; and in PSII-enriched membrane fragments that contain PTOX [49–50]. A precedent for ROS signaling comes from the well-known singlet oxygen retrograde pathway [51–52].

Although we recognize that high concentration of ROS are toxic (and might explain the phenotype of plastids in the white cells), our observation that white cells produce ROS is consistent with the idea that ROS signaling might play a role in coordinating developmental, physiological and biochemical processes in *im* leaves. It is also well-established that ROS mediate signaling pathways initiated in response to pathogen invasion [53–55]. In this context it is interesting that *MEKK1* is the only diagnostic defense gene that is significantly upregulated in the white tissues. This might be because it is the only one directly responsive to ROS; we do not know whether *MEKKI* upregulation has any physiological significance in the white sectors. On the other hand, a coordinated upregulation of *MEKK1*, *EDS1* and *PAD4* in infected *im* white tissues is indicative of their ability to respond to pathogen attack: *EDS1* and *PAD4* are induced by MEKK1 [56], and they form a complex in both the nucleus and cytoplasm [57] that provides a basal immune response. In this context it is noteworthy that pathogenesis-related defense genes (*PR1*, *PR5* and *bG2*) do not respond to pathogen infection at wild type levels in white sector tissues. This finding is striking, and might perhaps be related to the significant cell wall remodeling that occurs during the development of the green and white sectors.

### Cell wall remodeling

Glucose is the main structural constituent of cellulose and hemicellulosic xyloglucan, while mannose is a constituent of the backbone of hemicellulosic polysaccahride galactomannan, a subsidiary component of cell walls. Galactose is present in the side chains of galactomannan and its reduction correlates to a decrease in mannose content (Table 1). This reduction might be caused by alterations in pectic rhamnogalacturonan side chains, since galactomannans are not widely present in common plant cell walls, such as rhamnogalacturonans. In addition, pectins are known to be targeted by plant pathogens’ hydrolases to accelerate infection processes [58]. Reductions in fucose might also reflect a reduction of galactose in xyloglucan where the disaccharide Gal-Fuc forms a side chain attached to a xylosilated backbone. Considered together, these data suggest that the biosynthesis of galactomannans and decoration of xyloglucan are affected in the *im* white sectors.

Lignin and cellulose form the primary mechanical barriers preventing pathogen invasion [59–60]. Lignin is composed of polyphenol residues and not polysaccharides; however, the lignin biosynthesis phenylpropanoid pathway involves the sequential conversion of the amino acid phenylalanine into sinapoylglucose [61]. Since mutations affecting sinapoylglucose accumulation in Arabidopsis cells also exhibit reduced lignin deposition, it correlates with our finding of decreased lignin content in white sectors of *im* [62]. A reduction of lignin and cellulose microfibrils (Fig 5A) together with modification of some hemicellulosic polysaccharides in the cell wall causes, perhaps, rearrangement of the whole cell wall structure, thereby increasing the lytic efficiency of bacterial hydrolases directed to the plant cell wall as well as the penetration and infection efficiency of *P*.*syringae* cells.

## Conclusions

In summary, we propose that tissue-specific alterations in cell wall composition in *im* are a reflection of alterations in gene expression that remodel leaf development in response to oxidative stress and that also promote sink-source interactions between the green and white sector, both of these efforts are initiated very early in leaf development. According to this scenario, enhanced pathogen susceptibility in the white sectors is a secondary consequence of this development reprogramming. If so, it is not clear why some defense pathways are operational in the white sectors and others are not (pathogen related genes), although considerable cross talk between pathways can be envisioned in fashioning each tissue-type. Regardless, we conclude that both a lack of activation of pathogen related genes and modification of cell wall composition are a consequence of impaired chloroplast biogenesis in im, and that these result in compromised resistance to pathogen invasion. This conclusion is consistent with previous observations showing that alterations in cell wall composition lead to changes in plant signaling mechanisms and response to environmental conditions including phytopathogen invasion [37–40]. We conclude that *im* offers a novel model system to explore leaf developmental programming, cell wall metabolism and pathogen response.

## Supporting Information

S1 Table List of primers used in this work (5’–3’). (DOCX)
