# Supplementary material for: Impaired Chloroplast Biogenesis in Immutans, an Arabidopsis Variegation Mutant, Modifies Developmental Programming, Cell Wall Composition and Resistance to Pseudomonas syringae
Source: PLoS One. 2016 Apr 6;11(4):e0150983. doi: 10.1371/journal.pone.0150983 (PMC4822847; doi:10.1371/journal.pone.0150983)
Supplement: S1 Table — (DOCX) [file pone.0150983.s001.docx]

**Supplementary Data 1. List of primers used in this work (5’ – 3’):**

MEKK1 primers for qPCR: TATATGGAGCCTCGGGTGCA and CAGCAGTTCAGCTGCAGTT

PR1 primers for qPCR: TCTAAGGGTTCACAACCAGG and CCTTCTCGCTAACCCACATG

PR5 primers for qPCR: GAGGATCGGGAGATTGCAAA and GTCAGGGCAAGCGTTCTTGA

bGS2 primers for qPCR: AGCCGTTGATACTGTCATGT and GAATATTTGCAGCTTCTCCC

EDS1 primers for qPCR: CTGGTACAGTCGATGGGAAAG and TCTTCTATCCATGCTAGTTTC

PAD4 primers for qPCR: TTGCCAGTCACCGGAGATGT and GAGATAGAAGCCAAAGTGCGG

At Chloroplast DNA primers for qPCR: CGTGGAATTCGTTGAGAGTTAG and GATCGACTAACATCACGGAAAG

Actin2 primers for qPCR: GAAACCCTCGTAGATTGGCA and CTCTCCCGCTATGTATGTCGC
